# 3D-Printed Gastroretentive Sustained Release Drug Delivery System by Applying Design of Experiment Approach

**DOI:** 10.3390/molecules25102330

**Published:** 2020-05-16

**Authors:** Hyeon Myeong Jeong, Kwon-Yeon Weon, Beom Soo Shin, Soyoung Shin

**Affiliations:** 1 School of Pharmacy, Sungkyunkwan University, Suwon, Gyeonggi 16419, Korea; wise219143@skku.edu; 2 College of Pharmacy, Daegu Catholic University, Gyeongsan, Gyeongbuk 38430, Korea; weonky@cu.ac.kr; 3 College of Pharmacy, Wonkwang University, Iksan, Jeonbuk 54538, Korea

**Keywords:** 3D printing, drug delivery system, floating system, sustained release, baclofen, design of experiments

## Abstract

This study aimed to develop a novel oral drug delivery system for gastroretentive sustained drug release by using a capsular device. A capsular device that can control drug release rates from the inner immediate release (IR) tablet while floating in the gastric fluid was fabricated and printed by a fused deposition modeling 3D printer. A commercial IR tablet of baclofen was inserted into the capsular device. The structure of the capsular device was optimized by applying a design of experiment approach to achieve sustained release of a drug while maintaining sufficient buoyancy. The 2-level factorial design was used to identify the optimal sustained release with three control factors: size, number, and height of drug-releasing holes of the capsular device. The drug delivery system was buoyant for more than 24 h and the average time to reach 80% dissolution (T_80_) was 1.7–6.7 h by varying the control factors. The effects of the different control factors on the response factor, T_80_, were predicted by using the equation of best fit. Finally, drug delivery systems with predetermined release rates were prepared with a mean prediction error ≤ 15.3%. This approach holds great promise to develop various controlled release drug delivery systems.

## 1. Introduction

Solid oral dosage forms such as tablets and capsules are the most preferred drug formulations in the current market. The popularity of oral dosage forms is associated with its advantages of an accurate unit dose, stability, ease of handling, and high patient compliance compared to other drug formulations. However, traditional immediate release (IR) oral formulations present high peak-to-trough fluctuations and need frequent doses, i.e., administrations several times a day to maintain the therapeutic concentration. Thus, they often lead to extend the periods of toxicity or ineffectiveness, and discourage patient compliance.

Sustained release (SR) oral drug delivery systems are intended to achieve stable plasma drug concentrations within the desired range for an extended period of time [1], thereby overcoming the limitations of conventional IR formulations. By maintaining the therapeutic plasma concentrations, SR formulations may require less frequency of administration, which leads to improved patient compliance, minimized unwanted side effects, and maximized therapeutic efficacy [2]. Thus, the ideal drug delivery system for SR formulations should be inert, biocompatible, mechanically robust, comfortable for the patient, capable of achieving high drug loading, safe from accidental release, simple to administer and remove, and easy to fabricate and sterilize. 

The recent advances in oral SR technology are attributed to the development of novel biocompatible polymers and machinery that allow the preparation of innovative dosage forms in a reproducible manner [3,4]. Matrix technologies have proven to be popular among oral sustained drug delivery technologies because of their ease of manufacturing and simplicity of formulation, a high degree of reproducibility, the stability of the excipients and dosage form, and ease of technology transfer during scale-up and process validation. Hydrophilic matrix systems are widely used for providing a sustained release from the solid oral dosage forms. Hydroxypropyl methylcellulose (HPMC), a semisynthetic polymer derived from cellulose, shows minimal interaction problems when used in basic, acid, or other electrolytic systems due to its nonionic nature and has been used as one of the most popular drug release rate modifiers. HPMC is also enzyme resistant, chemically stable over a wide pH range, and has consistently high quality and regulatory approval, making it an excellent carrier material for a matrix system [2,3,5,6,7].

However, SR formulations taking advantage of biocompatible polymers and machinery usually require a high cost of formulation as well as long development time. The performance of SR formulations depends on the drug release from the formulation that is controlled by the unique properties of the drug, polymer, and the interaction between the drug and polymer. Thus, a new formulation should be carefully designed and prepared for each drug, which may be achieved after multiple trials and errors. 

Moreover, the development of a conventional SR formulation may not be feasible for certain drugs [6,7]. For drugs that (1) are locally active in the stomach such as ranitidine and amoxicillin, (2) have an absorption window in the stomach or in the upper small intestine such as riboflavin and baclofen, (3) are unstable in the intestinal or colonic environment such as verapamil and captopril, or (4) exhibit low solubilities at high pH values such as ofloxacin and cinnarizine, the benefit of SR could not be achieved by using only SR technology [6,7,8,9]. The formulation for these drugs should, therefore, not only be able to release the drug in a sustained manner but also remain in the stomach in order to achieve the therapeutic advantages of SR. 

Among the various methods applied for gastroretentive dosage forms, the floating system is one of the most practical and widely explored mechanisms. The buoyancy allows the system to remain in the stomach for a prolonged period and the drug is released at the desired rate from the system [10]. However, the floating property and release kinetics of the most available gastroretentive formulations are dependent on each other. That is, the floating properties of the dosage form may decrease as the drug release progresses, which may lead to premature evacuation before complete drug release [11]. Therefore, it is necessary to separate the sustained release and floating properties from each other [12,13].

Three-dimensional (3D) printing or additive manufacturing is a powerful technology that can fabricate complex 3D objects accurately and precisely from computer aid design. The unique advantages of 3D printing technology, including rapid prototyping, have shown its potential to improve upon current pharmaceutical dosage forms through complex and customized dosage forms which are not cost-effective or otherwise impossible [14,15]. For example, various innovative oral dosage forms such as a multi-layered polypill [16], a floating pulsatile drug delivery system [17], and an on-demand fully customizable drug tablet [18] have been explored. In our previous work, we also have shown the potential utility of 3D printing technology to design a gastro-floating drug delivery device combined with a conventional SR tablet for extended gastric residence time to improve oral bioavailability [13].

In the present study, we aimed to design and develop a novel drug delivery system by using a capsular device that can be floating in the gastric fluid and control the drug release rate from the inner IR tablet. The abilities to control the buoyancy and drug release rate are attributed to the structure of the capsular device so that a conventional IR tablet could be simply inserted in the device. A commercial baclofen IR tablet, a centrally acting skeletal muscle relaxant, was used as a model drug to examine the feasibility of the present drug delivery system for sustained drug delivery. The strength of 3D printing in rapid prototyping allowed us to efficiently prepare various capsular devices with different structures to achieve sustained drug release. The design of experiment (DoE) approach was also applied to efficiently optimize the capsular device.

## 2. Results

### 2.1. Floating Ability and Operation of the Drug Delivery System

**Figure 1:**
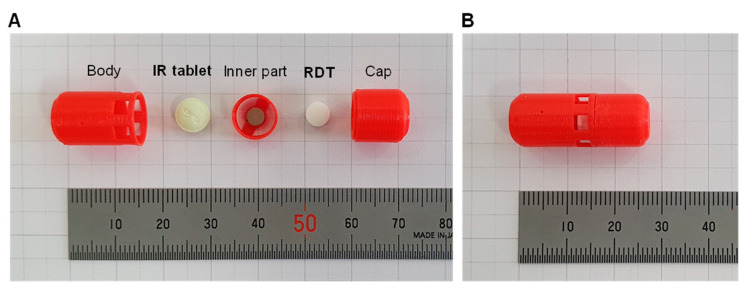
Components of the gastroretentive sustained release drug delivery system, i.e., 3D-printed capsular device, RDT, and IR tablet (**A**) and a final drug delivery system after assembly (**B**).

**Figure 2:**
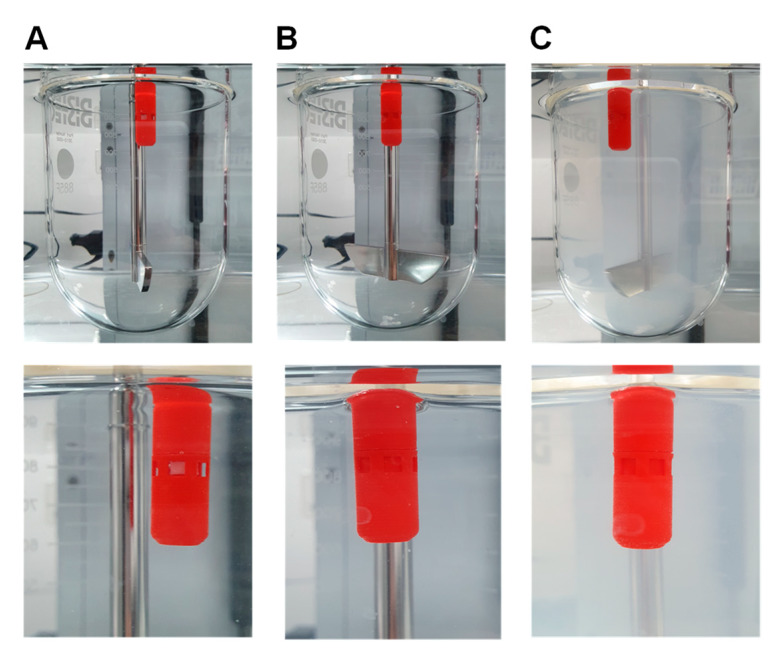
Gastroretentive sustained release drug delivery system immediately after putting in the dissolution with the windows open, i.e., open phase (**A**), with the windows closed after the RDT disintegrated, i.e., closed phase (**B**), and after the completion of drug release (**C**).

Figure 1 shows the components of the gastroretentive sustained release drug delivery system, i.e., 3d-printed capsular device, RDT, and IR tablet before assembly, and a final drug delivery system after assembly. The floating ability and operation of the prepared drug delivery system were evaluated by the in vitro dissolution test. Once put in the dissolution medium, the drug delivery system was floating in the upright position. Figure 2A shows the drug delivery system with the windows open, i.e., open phase, immediately after put in the dissolution medium. In the dissolution medium, the RDT disintegrated within 1 min and the drug delivery system closed windows, i.e., closed phase (Figure 2B). Then, baclofen from the inner IR tablet was gradually released through the drug-releasing holes while the drug delivery system remains buoyant (Figure 2C).

The drug delivery systems with the air pocket inside the cap were buoyant in the dissolution medium for more than 24 h. The floating abilities were not different for various drug delivery systems regardless of the size, number, and height of the drug-releasing holes. Meanwhile, the devices also maintained durability. One the other hand, the reference device without an air pocket immediately sank to the bottom of the dissolution tester.

### 2.2. In Vitro Dissolution of a Factorial Design Batch

**Figure 3:**
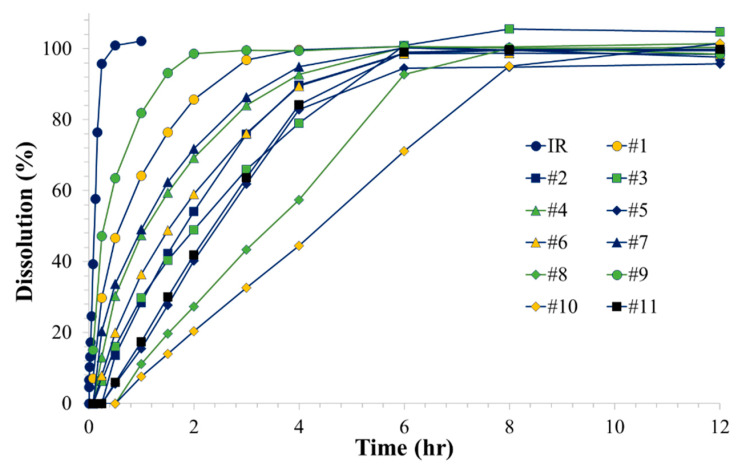
In vitro dissolution profiles of the gastroretentive sustained release drug delivery systems in a factorial design batch.

Drug release profiles of the drug delivery systems that were designed by two-level factorial experimental design were determined by the in vitro dissolution test. Figure 3 shows the obtained individual drug release profiles of 11 drug delivery systems and the intact baclofen IR tablet. Complete dissolutions were observed from all the tested drug delivery systems without significant lag time (Figure 3). The determined T_80_ values are summarized in Table 1. While the T_80_ of a baclofen IR tablet itself was less than 11.6 min, the T_80_ of the commercial baclofen IR tablet in the different drug delivery systems ranged from 0.95 to 6.74 h. The three drug delivery systems, i.e., No. 2, 3, and 6, which are the triplicate of a center point in the design space, would theoretically present the identical T_80_, but result in different T_80_ values, which is due solely to experimental error. From the repeated runs of the center point, the experimental error could be estimated and reflected in the model [19].

### 2.3. Effect of Control Factors on the Response (T_80_)

A statistical model incorporating polynomial and interactive terms was derived to describe the effects of control factors, i.e., the size, number, and height of the drug-releasing holes in the capsular device, on the response. i.e., T_80_. The obtained mathematical model in coded terms is described as follows:

T_80_ = 3.48 − 0.506·x_1_ − 0.306·x_2_ + 1.47·x_3_ + 0.471·x_1·_x_2_ + 0.156·x_1·_x_3_ − 0.078·x_2·_x_3_ + 0.617·x_1·_x_2·_x_3_ (1)

where x_1_, x_2_, and x_3_ are the size, number, and height of the drug-releasing holes of the capsular device, respectively. Table 2 summarizes the results of ANOVA for the experimentally observed response (T_80_). The model terms including the size (x_1_), height of the drug-releasing holes (x_3_), the interaction of the size and number (x_1·_x_2_), and the interaction among the three factors (x_1·_x_2·_x_3_) found to significantly affect the response, i.e., T_80_. Notably, the increase of the height, x_3_, increased T_80_ as indicated by the relatively higher positive coefficient in Equation 1. Curvature appeared to be insignificant, and the actual model *R*^2^, predicted *R*^2^, and adjusted *R*^2^ were 98.24%, 75.90%, and 94.15%, respectively.

**Figure 4:**
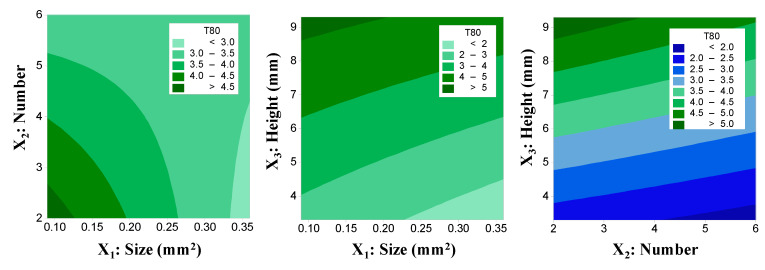
The effects of the control factors on T_80_ of the gastroretentive sustained release drug delivery system in contour plots.

Figure 4 shows the effects of the control factors on T_80_ in the contour plots. T_80_ increased significantly with the increase in the height of the drug-releasing holes, while the size and number of drug-releasing holes had a marginal effect. 

### 2.4. Optimization of the Drug Delivery System 

Finally, based on the derived mathematical relationship between the control factors and response (T_80_), three types of drug delivery systems that have fast (T_80_ = 2 h), medium (T_80_ = 4 h), and slow (T_80_ = 6 h) drug release rates were designed by optimal solutions. The optimal settings for each drug delivery system to achieve T_80_ of 2, 4, and 6 h are summarized in Table 3. 

**Figure 5:**
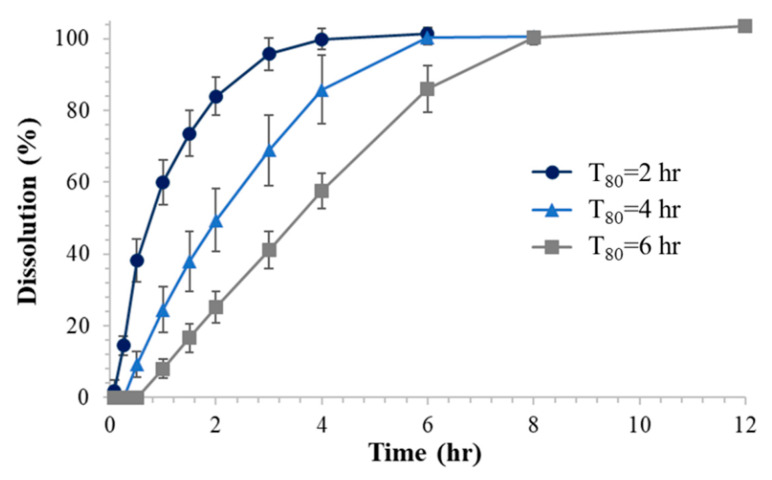
In vitro dissolution profiles of the optimized gastroretentive sustained release drug delivery systems with the target T_80_ of 2, 4, and 6 h (*n* = 3).

The three types of gastroretentive sustained release drug delivery systems were then evaluated by the in vitro dissolution test and the obtained dissolution profiles are depicted in Figure 5. The observed T_80_ values in comparison with the predicted values are shown in Table 3. The mean absolute errors (MAE) of T_80_ from the optimized drug delivery systems compared to the predicted values were less than 0.60 h. The mean absolute percentage error (MAPE) was from 7.2% to 21.6%.

## 3. Discussion

A novel gastroretentive sustained release drug delivery system that consisted of a 3D-printed capsular device, rapid dissolving tablet (RDT), and a drug-containing immediate release (IR) tablet was developed and optimized to achieve sustained drug release. The drug delivery system was designed to control the opening and closing of the windows by the magnetic attraction and RDT dissolution, which allows the initial water influx into the capsular device. Once it contacts the gastrointestinal fluid, the water comes into the capsular device through the open windows in the initial open phase (Figure 2A). Then, the RDT is immediately disintegrated within 1 min by the water influx and the inner part of the device moves upward by the magnetic attraction and closes the windows (Figure 2B). In this closed phase, the drug delivery system could release the drug from the IR tablet via the drug-releasing holes. Since the drug release from the drug delivery system is mediated by the drug-releasing holes, the modulation of the drug-releasing holes allowed control of the drug-releasing rate from the inner IR tablet. Meanwhile, the drug delivery system was designed to remain buoyant with an upright position, which is attributed to the air pocket inside the cap.

Our data showed that the developed drug delivery system could successfully modulate the drug-releasing rate from the inner IR tablet by the structural modification of the capsular device. Drug release rate of a model drug, baclofen, varied depending on the geometry of the capsular device, i.e., size, number, and height of the drug-releasing holes (Figure 3, Table 1). Notably, as the height of the drug-releasing holes increases, the dissolution time of baclofen significantly increases. Based on the designed experiment, a mathematical relationship between the structure of the drug delivery device, i.e., the size, number, and height of the drug-releasing holes, and the dissolution rate represented by T_80_ was derived. Finally, three types of gastroretentive sustained release drug delivery systems with the predetermined T_80_ of 2, 4, and 6 h were successfully prepared with the mean absolute error of less than 0.6 h.

In this study, the strength of 3D printing in rapid prototyping allowed efficient preparation of various capsular devices with different structures generated by design of experiment (DoE). In addition to its potential utility for printing dosage forms, there has been a growing interest in the application of 3D printing technology for the development of drug delivery devices with modified release characteristics using computer-aided design (CAD) [20]. For example, a wearable personalized oral delivery device [21] and gastroretentive drug delivery systems that combine a drug delivery device and conventional tablets [12,13] have been reported. The present drug delivery system also combined a functional drug delivery device with a commercial IR tablet and achieved sustained drug release at a predetermined rate as well as buoyancy. Although previously reported floating devices also affected the drug release rates, they still relied on the drug–polymer interaction to control drug release rates to achieve sustained release [12,13]. Thus, the primary advantage of the present sustained release drug delivery system is that a commercial IR tablet could be simply placed in the device without further formulation effort. Compared to the conventional approach to develop SR formulations relying on polymers, the present approach may be easily applied to various drugs by simply using their IR product, which in turn would save time and cost for the formulation development.

Another advantage of the present approach is that the capsular device provides both sustained drug release rates and buoyancy. Thus, this approach is applicable for various drugs where simple SR formulations do not result in the improvement of bioavailability and therapeutic efficacy due to their physicochemical properties, absorption characteristics, or the site of action. For example, the model drug of the present study, baclofen, is a Biopharmaceutical Classification System (BCS) class III drug with high solubility and low permeability. The presence of an absorption window for baclofen in the gastrointestinal tract has been indicated [2,22,23,24]. Due to the absorption window, conventional SR tablets that are intended to continuously release a drug while passing through the gut may not improve the oral bioavailability. Indeed, the reduced in vivo oral bioavailability of the SR formulations with the slower drug release rates have been observed for baclofen [2] as well as other drugs with an absorption window including acyclovir and loxoprofen [6,7]. Thus, gastroretentive sustained release formulations, which could remain in the stomach during the drug release, have been explored to improve the oral bioavailability of baclofen [25,26,27]. By providing sufficient buoyancy during the drug release, the present gastroretentive sustained release drug delivery system may be applied for various drugs like baclofen to improve the oral bioavailability as well as control drug release rates. 

Furthermore, the two functions of the present drug delivery system, i.e., buoyancy and the modification of drug release rate are independent, which presents an additional strength compared to other floating gastroretentive systems. Gastro-floating systems including hydrodynamically balanced systems, raft forming systems, and gas-generating systems represent one of the widely investigated mechanisms to develop gastroretentive formulations [28,29]. However, a major limitation of these conventional floating systems is that gastric retention is not controlled by its floating mechanism alone [29]. For example, drug release from a hydrodynamically balanced system is controlled by diffusion and erosion of the gel barrier that also produces a floating mass. The gel polymer barrier may not provide sustained buoyancy until the complete drug release [28]. The changes in the floating strength with time, therefore, may lead to premature evacuation before the complete drug release. In this drug delivery system, however, the floating ability and drug release are separated from each other. While the buoyancy contributes to the air pocket inside the cap, the drug release rate is controlled by the variation of the drug-releasing holes of the capsular device. Thus, they could be optimized independently to provide sufficient buoyancy until the drug release is completed. As a result, the immediate and sustained buoyancy was identical for the various capsular devices with different release rates. The drug delivery system also maintained buoyancy regardless of the presence of the baclofen IR tablet, as well as after the drug was completely dissolved (Figure 2C).

During the development, we also applied a DoE approach, which allowed efficient identification and optimization of the factors affecting the drug release rates. By using a 2-level factorial design, it was possible to determine the critical factors that control the performance of the sustained release drug delivery system and derive a mathematical model to describe the effects of the selected factors on the response. Therefore, the optimal structure of the capsular device to attain a specific release rate could be developed. Initially, it was assumed that the total area and location of the drug-releasing holes might affect the drug release rate. Since the size of a single drug-releasing hole is limited by the need to hold the IR tablet before its complete dissolution, the total area could be manipulated by the number and individual size of the holes. Thus, the three control factors, i.e., the size, number, and height of drug-releasing holes were selected and explored to modulate the drug release rate from the capsular device. Preliminary studies also found that drug release tends to be faster as the total area, i.e., the size and number, of the drug-releasing holes increases as expected. The drug release rate also increased as the drug-releasing holes are put close to the bottom of the device. Since the inner IR tablet was placed in the bottom of the capsular device, the dissolved drug should migrate from the IR tablet to the holes to be released out of the drug delivery system. Thus, it was postulated that the higher the height of the drug-releasing holes, the slower the drug release rate becomes. As expected, the obtained mathematical model (Equation (1)) suggested that the size (x_1_) and number (x_2_) decrease T_80_, while the height (x_3_) increases T_80_. It is also indicated that the effects of size (x_1_) and height of the drug release holes (x_3_), and the interaction between size and number (x_1_·x_2_) on T_80_ were significant (*p* < 0.05, Table 2).

In our previous study, the abdominal X-ray images showed that a 3D-printed gastroretentive system with a similar air pocket structure remained in the stomach for more than 12 h and was excreted at 48 h after oral administration in a Beagle dog [13]. Although the in vivo performance of the present drug delivery system needs to be examined in further studies, the present system may also be able to be maintained in the stomach for a sufficient time for complete drug release and be excreted by normal gastrointestinal motility. In order to control the excretion of the gastroretentive system, the gastroretentive device could be printed in part with another material that is biodegradable or has the ability to lose its integrity after a desired time period. For example, since polyvinyl alcohol (PVA) is dissolved in the water, the air pocket structure composed of PVA would disappear as PVA dissolves. As the air pocket disappears, the gastroretentive system would lose its buoyancy and be removed from the stomach. In addition, the closed length of the final drug delivery system is approximately comparable to the size 00 capsule, which is the largest capsule used for human oral preparation. Thus, the size of the drug delivery system needs to be reduced for further development.

In summary, we have successfully demonstrated the potential of a novel sustained release drug delivery system that combined a capsular device with a commercial IR tablet of baclofen for sustained drug delivery. This approach may be applied to modify drug release rates of other conventional formulations without complex formulation efforts. 3D printing technology allowed efficient and straightforward production of the prototypes of the device with various architectures and optimization of its structure for the intended release rate. By combining a functional drug delivery device with conventional oral formulations, other forms of novel sustained release formulation could also be developed to overcome limitations of traditional formulations.

## 4. Materials and Methods 

### 4.1. Chemicals and Reagents

A commercially available IR tablet of baclofen, Prex tab^®^ 10 mg was purchased from Korea United Pharm Inc. (Seoul, Korea). Lactose (Flowlac^®^ 100) was obtained from Meggle Pharma Co. (Wasserbug, Germany) and magnesium stearate was purchased from Faci Asia Pacific Pte Ltd. (Jurong Island, Singapore). Sodium hydroxide and sodium chloride were obtained from Samchun Chemical Co., Ltd. (Seoul, Korea). Baclofen was purchased from Sigma-Aldrich Co. (St. Louis, MO, USA). Ethanol, hydrochloric acid, phosphoric acid, and sodium dihydrogen phosphate were the products of Merck Co. (Darmstadt, Germany). High performance liquid chromatography (HPLC) grade methanol and water were purchased from J.T. Baker Co. (Philipsburg, NJ, USA). Polylactic acid (PLA) filament was obtained from Raise3D, Inc. (Irvine, CA, USA).

### 4.2. Preparation of a Rapidly Dissolving Tablet

The rapidly dissolving tablet (RDT), which was designed to disintegrate faster than the drug-containing IR tablet does, i.e., within 1 min, consisted of 99.5% lactose (Flowlac^®^ 100) and 0.5% magnesium stearate. RDT was prepared by direct compression and compressed by 0.3-ton pressure using a manual single punch tablet press (KTP-05, Koreamedi Co., Ltd., Daegu, Korea) with a round-shaped punch (diameter: 5 mm). 

### 4.3. Design of the Capsular Device

**Figure 6:**
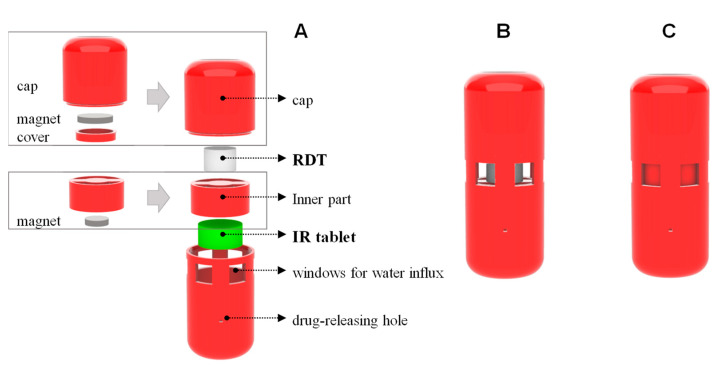
Structure of the 3d-printed gastroretentive sustained release drug delivery system that is composed of a cap with an air pocket inside and a body with windows for water influx and drug-releasing holes before assembly (**A**) and a final drug delivery system after assembly with the windows opened (**B**). After a rapidly dissolving tablet is dissolved, the windows for water influx are closed (**C**).

The drug delivery system was designed to combine a capsular device and RDT, and an IR tablet, in order to control the drug-releasing rate from the inner tablet with buoyancy in the gastrointestinal fluid. The structure of the capsular device was designed by the computer-aided design (CAD) software, Rhino 6 (Robert McNeel & Associates, Seattle, WA, USA). Figure 6A shows the components of the gastroretentive system before assembly, i.e., the capsular device that consists of a cap, inner part, and body, RDT, and IR tablet. The cap contains a cavity inside for buoyancy and a magnet attached to the bottom of the cap. The body was designed to have a space to contain a baclofen IR tablet and have six large windows for initial water influx and various small holes for drug release on its wall. The size, number, and height of the drug-releasing holes were varied to obtain specific drug release rates. An open-ended hollow cylinder-shaped inner part with an embedded magnet on its top was intended to move up and down by the magnetic attraction after inserted in the body. 

For assembly, a commercial baclofen IR tablet was inserted in the body first and the inner part was put on the top of the baclofen IR tablet. An RDT was then inserted between the inner part and cap as a prop. Due to the attraction between magnets embedded on the bottom of the cap and top of the inner part, the inner part tends to move up and attach to the cap, which was prevented by the prop. Once assembled, therefore, the windows are opened and provide a waterway for water influx from outside to the baclofen IR tablet in the capsular device until the RDT disintegrates (Figure 6B). Once the drug delivery system is put in the gastrointestinal fluid, the RDT immediately disintegrates, and the inner part moves towards the cap and closes the windows (Figure 6C). The baclofen IR tablet inside the body of the capsular device then dissolves by the water influx through the windows while the windows are opened, and releases drug through the drug-releasing holes after the windows are closed.

### 4.4. Preparation of the Drug Delivery System

The capsular device was printed with a PLA filament using a fused deposition modeling (FDM) 3D printer (Raise3D N2, Raise3D, Inc., Irvine, CA, USA). After each part of the device was printed, magnets were embedded in the cap with the inner part first (Figure 6A). Then, a commercial baclofen IR tablet (Prex tab^®^ 10 mg, Korea United Pharm. Inc., Seoul, Korea) was inserted in the capsule body and the magnet was embedded and the inner part was placed on the top of the IR tablet in the body. After an RDT was also placed on the top of the inner part, the cap was finally closed (Figure 6B). Since the full circumferences of the cap and body have a locking ring structure, the cap and body interlocked to form a secure closure.

### 4.5. Experimental Design

A two-level factorial design was generated by Minitab^®^ 18 software (Minitab, LLC., State College, PA, USA) to derive a mathematical model for the optimized drug delivery system. The factorial design batch consisted of eight factorial points (2^3^) and one center point. The triplicate mid-level of the variables, i.e., the center point, was included to estimate experimental error for an improved statistical significance. Therefore, a total of 11 experiments (11 = 2^3^ + 1 × 3) were carried out to examine the main effect of three control factors: x_1_ (size of the drug release holes), x_2_ (number of the drug release holes), x_3_ (height of the drug release holes from the bottom). The time required for 80% dissolution (T_80_) was selected as a response. Table 1 summarizes the experimental runs with three control factors, i.e., independent variables, and a response, i.e., the dependent variable. The drug delivery system No. 2, 3, and 6 are the triplicate of a center point. The values of x_1_, x_2_, and x_3_ were selected in the range of 0.09 to 0.225 mm^2^, 2 to 6, and 3.3 to 9.3 mm, respectively. 

To validate the derived model, three types of drug delivery systems were designed by optimal solutions to achieve T_80_ of 2, 4, and 6 h. By comparing the predicted and observed T_80_ values, the mean absolute error (MAE) and mean absolute percentage of error (MAPE) were calculated:

$$\mathrm{MAE}=\frac{1}{n}\cdot \sum^{\text{​}}\left|Observed-Predicted\right|$$

$$\mathrm{MAPE}=\frac{1}{n}\cdot \sum^{\text{​}}\left|\frac{Observed-Predicted}{Observed}\right|\times 100$$

### 4.6. In Vitro Tests

The floating ability and drug release rates of baclofen from the prepared drug delivery systems were determined by the paddle method using the Distek Dissolution System 2500 coupled with the Evolution Dissolution Sampler 4300 (Distek, North Brunswick, NJ, USA). The dissolution test was performed using 900 mL of 0.1 N HCl buffer (pH 1.2) as a dissolution medium at 37 ± 0.5 °C. The paddle stirring speed was fixed at 100 rpm. The samples were collected at the predetermined time, and the medium was replaced with fresh medium. The collected samples were filtered through a 45 μm polyethylene syringe filter (Distek) and immediately analyzed by the HPLC method.

### 4.7. Drug Analysis

Baclofen concentrations in the dissolution medium were determined by HPLC using a Waters Alliance 2695 coupled with the Waters UV detector 2487 (Waters, Milford, MA, USA). Baclofen was separated on a Zorbax 300SB-C18 (250 × 4.6 mm, i.d., 5 μm, Agilent, Santa Clara, CA, USA). An isocratic solvent system consisting of sodium phosphate buffer (10 mM, pH 2.6) and methanol (70:30 *v/v*%) was used as the mobile phase at a flow rate of 1 mL/min. The column oven temperature was set at 30 °C and the total run time was 6.0 min. The sample injection volume was 20 μL and baclofen was detected at 220 nm. The working standard solutions for HPLC analyses were prepared by serial dilutions of the stock solution in the mobile phase at concentrations of 0.5, 1, 2, 5, 10, and 20 μg/mL.

### 4.8. Statistical Analysis

The two-level factorial design was employed to study the relationship between the independent variables and the dependent variable. The analysis of variance (ANOVA) was performed and a *P*-value less than 0.05 was evaluated to determine the significance of each term. Similarly, a lack of fit of the suggested model was also assessed. To determine the fitting extent of experimental data, the regression coefficient (*R*^2^) and adjusted *R*^2^ were determined.

## Figures and Tables

**Table 1:** Two-level factorial design with three control factors and the experimentally determined response (T_80_).

No	Control Factors			Response
	x_1_: Size of the Drug Release Hole (mm^2^)	x_2_: Number of the Drug Release Hole	x_3_: Height of the Drug Release Hole (mm)	y: T_80_ (h)
1	0.36	2	3.3	1.69
2	0.225	4	6.3	3.29
3	0.225	4	6.3	4.09
4	0.09	2	3.3	2.73
5	0.36	2	9.3	3.87
6	0.225	4	6.3	3.29
7	0.09	6	3.3	2.56
8	0.36	6	9.3	5.28
9	0.36	6	3.3	0.95
10	0.09	2	9.3	6.74
11	0.09	6	9.3	3.80

**Table 2:** Summary of the results of ANOVA for the observed response (T_80_).

Response	Source	Sum of Squares	DF	Mean Square	*F*-Value	*p*-Value
T_80_	Model	25.11574	7	3.587962	23.97947	0.012
	$x_{1}$	2.04674	1	2.04674	13.679	0.034 *
	$x_{2}$	0.747528	1	0.747528	4.995965	0.111
	$x_{3}$	17.25884	1	17.25884	115.3462	0.002 *
	$x_{1}x_{2}$	1.774022	1	1.774022	11.85634	0.041 *
	$x_{1}x_{3}$	0.195791	1	0.195791	1.308532	0.336
	$x_{2}x_{3}$	0.049017	1	0.049017	0.327598	0.607
	$x_{1}x_{2}x_{3}$	3.043797	1	3.043797	20.34265	0.020 *
	Residual	0.448879	3	0.149626		
	Lack of Fit	0.02397	1	0.02397	0.112822	0.769
	Pure Error	0.42491	2	0.212455		
	Cor Total	25.56461	10			

*, *p* < 0.05.

**Table 3:** The optimal settings of control factors and the responses (T_80_) for drug delivery systems to achieve fast (T_80_ = 2 h), medium (T_80_ = 4 h), and slow (T_80_ = 6 h) release.

Type	Optimal Setting			T_80_ (h)			
	x_1_: Size of the Drug-Releasing Hole (mm^2^)	x_2_: Number of the Drug-Releasing Hole	x_3_: Height of the Drug-Releasing Hole (mm)	Predicted	Observed (*n* = 3)	MAE	MAPE (%)
fast	0.36	6	4.95	2.17	1.81 ± 0.29	0.36	21.6
medium	0.36	6	7.5	4.01	3.7 ± 0.65	0.60	17.0
slow	0.09	2	8.1	5.97	5.59 ± 0.42	0.38	7.2

MAE—mean absolute error; MAPE—mean absolute percentage error.
